# Ipilimumab-induced renal granulomatous arteritis: a case report

**DOI:** 10.1186/s12882-019-1552-2

**Published:** 2019-10-11

**Authors:** Mathilde Lemoine, Baptiste Dilly, Alexandre Curie, Vivien Hébert, Charlotte Laurent, Mélanie Hanoy, Steven Grangé, Dominique Guerrot, Arnaud François, Dominique Bertrand

**Affiliations:** 1 Nephrology department, Rouen University Hospital, 147 avenue du Maréchal Juin 76230 Bois Guillaume, Rouen, France; 2 Dermatology department, Rouen University Hospital, Rouen, France; 3 Medical Intensive Care Unit, Rouen University Hospital, Rouen, France; 4 INSERM U1096, Rouen University Medical School, Rouen, France; 5 Pathology department, Rouen University Hospital, Rouen, France

**Keywords:** Acute kidney injury, Renal granulomatous arteritis, Immune checkpoint inhibitors, Ipilimumab, Immune related adverse events

## Abstract

**Background:**

Immune Checkpoint Inhibitors (ICPIs) are promising new drugs in treatment of advanced tumours targeting cytotoxic T-lymphocyte antigen-4 (CTLA-4) and programmed cell death protein-1 (PD1) or its ligand (PDL-1). Ipilimumab is a monoclonal antibody targeting the CTLA-4 receptor used in treatment of metastatic melanoma. By increasing activity of the immune system, ICPIs lead to immune-related adverse events, such as dermatitis, colitis or hepatitis. ICPIs-related kidney adverse events are rare and acute tubulointerstitial nephritis with or without granuloma have mainly been reported.

**Case presentation:**

We report a case of acute kidney injury in a patient with melanoma treated by ipilimumab. Kidney biopsy revealed acute interlobular and juxtaglomerular granulomatous arteritis, which has not yet been reported in patients treated by ICPIs. Kidney function partially recovered after ipilimumab discontinuation and oral prednisone. Unfortunately, the patient died a few months later from progression of his melanoma.

**Conclusion:**

This case highlights a new mechanism of acute kidney injury related to ICPIs and supports the interest of kidney biopsy in case of ICPIs related acute renal failure.

## Background

Immune check point inhibitors (ICPIs) are monoclonal antibodies stimulating the immune system by blocking the coinhibitory receptors on T cells, leading to antitumoral response. This class of drugs includes cytotoxic T-lymphocyte antigen 4 (CTLA-4) inhibitors, such as ipilimumab, programmed cell death protein 1 (PD1) inhibitors and programmed cell death protein-ligand 1 (PDL-1) inhibitors. These molecules disrupt immune tolerance, leading to anti-tumor immunity but also inflammatory side effects, named immune-related adverse events (IRAEs), such as dermatitis, colitis, hepatitis or endocrinopathies [1].

ICPI-related acute kidney injuries are reported in 0 to 4% of treated patients [2]. The main pathologic lesion is interstitial nephritis, with or without granuloma [2, 3]. A few cases of immune glomerulonephritis have also been described [4, 5].

Here, we describe the first case of ipilimumab-induced renal granulomatous arteritis in a patient with melanoma.

## Case presentation

A 70-year-old man with anal metastatic melanoma was admitted on December 2017 for severe asthenia.

**Fig. 1:**
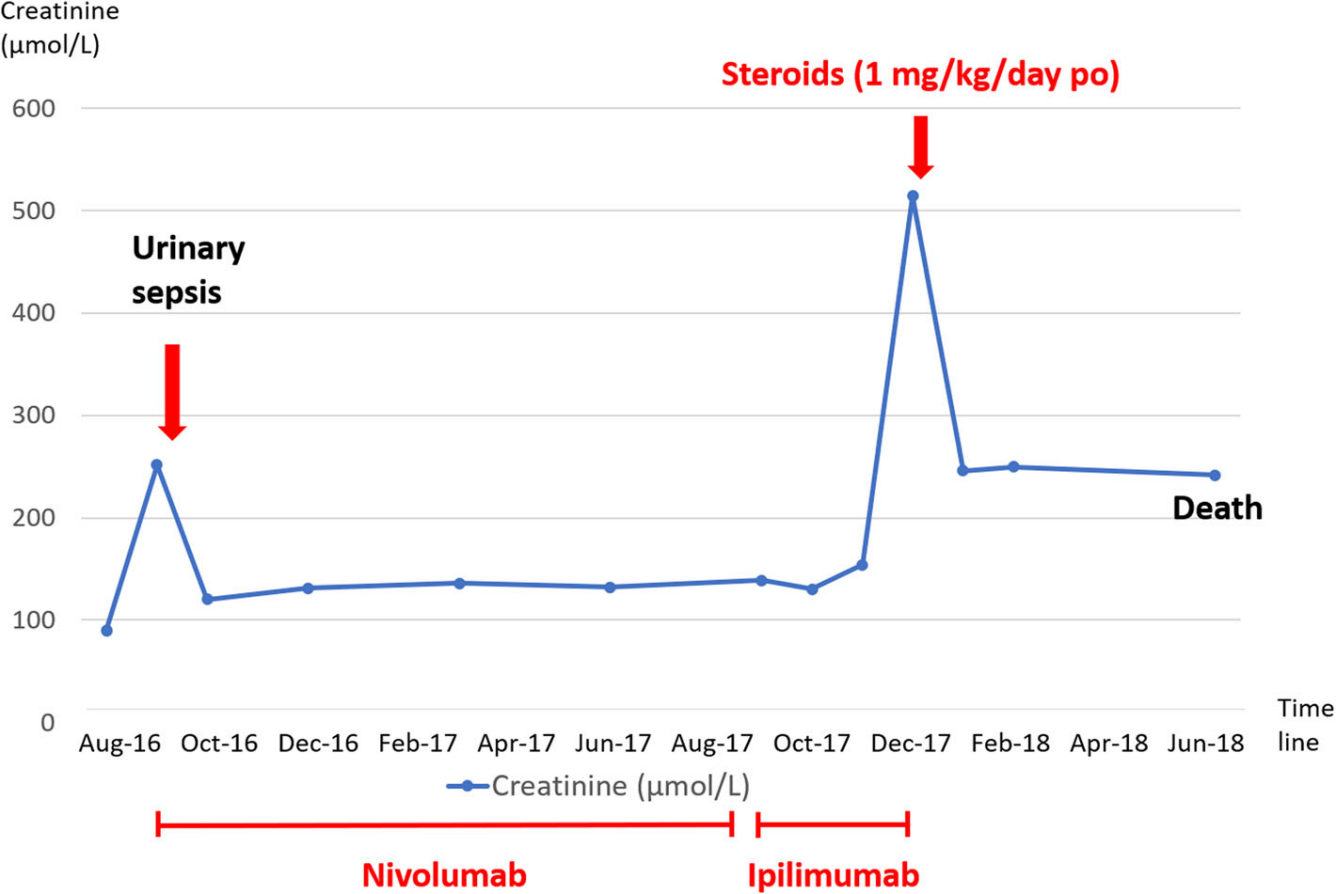
Evolution of kidney function

He was treated on August 2016 by abdominoperineal resection and bladder reconstruction. Serum creatinine was 1.0 mg/dl before the surgery. He presented an acute kidney injury in a context of urinary tract infection one month later (serum creatinine 2.9 mg/dl). Kidney function recovered partially (serum creatinine 1.4 mg/dl - Fig. 1). In September 2016, he received nivolumab, a monoclonal anti-PD-1 receptor antibody. In October 2017, progression of the disease was detected on a pelvic MRI and treatment was switched by ipilimumab, a monoclonal anti-CTLA-4 receptor antibody, 3 mg/kg every three weeks. Serum creatinine was then at 1.7 mg/dl. He had no other medical history nor took any other drugs.

Approximately ten days after completion of his third cycle, he presented with a history of one-week fatigue. At admission, blood pressure was 116/60 mmHg and physical examination revealed no particularity. Laboratory findings revealed an acute renal failure with serum creatinine rising to 5.8 mg/dl (Fig. 1), a 24-h urine total protein excretion of 1.0 g and an inflammatory syndrome. Urine cytology showed leukocytes (100/mm^3^) and red blood cells (50/mm^3^). Ultrasonography excluded urinary tract obstruction. All laboratory results are detailed in Table 1.

**Table 1:** Laboratory results

	30.12.17
Serum creatinine level (μmol/l)	514
Blood urea nitrogen level (mmol/l)	30.2
Hemoglobin (g/dl)	9.4
Calcemia (mmol/l)	2.09
Proteinuria (g/24 h)	1.1
Hematuria (/mm^3^)	50–100
Leukocyturia (/mm^3^)	100–1000
Angiotensin convertase enzyme (U/l)	45
Anti-PR3	< 2.3
Anti-MPO	< 3.2

**Fig. 2:**
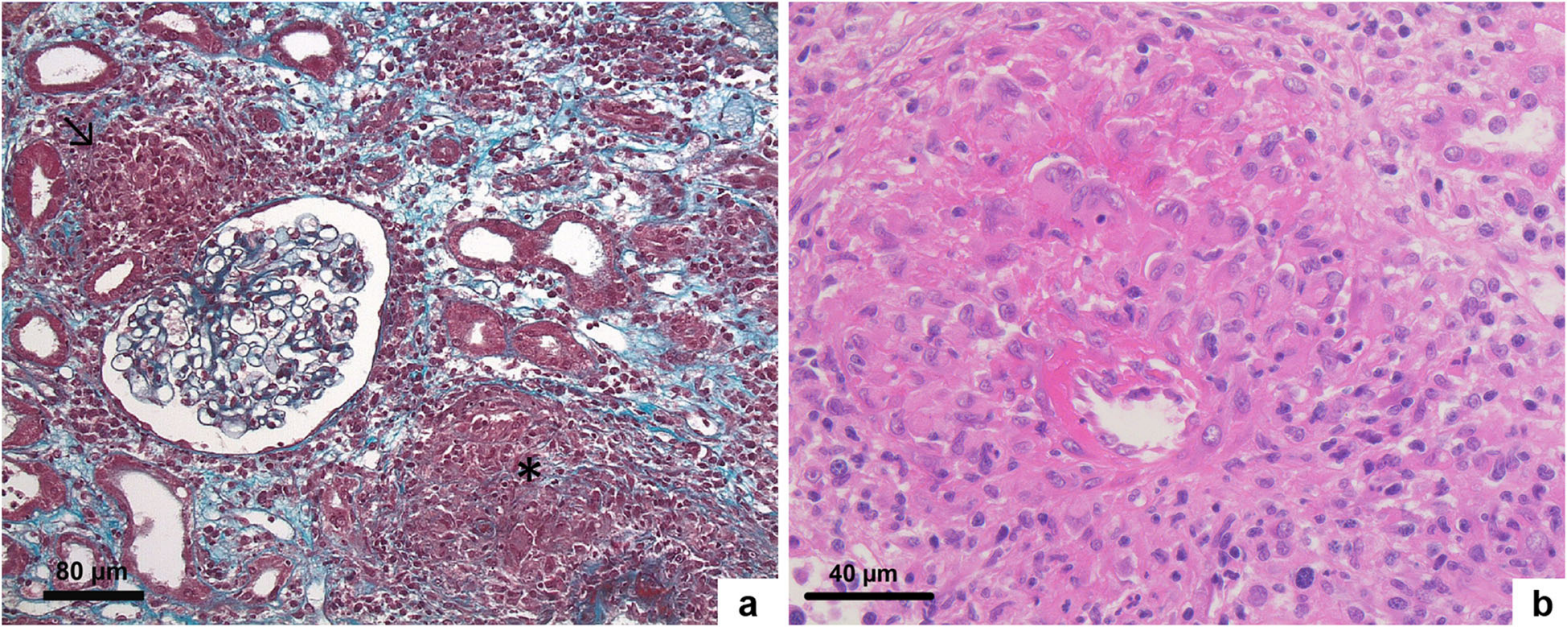
Kidney biopsy showing destruction of arteries by epithelioid cell granulomas (Masson’s trichrome (**a**) and H&E staining (**b**)). (**a-b**) Light microscopy. (**a**) Juxtaglomerular (↘) and periarteriolar (*) non caseating granulomas. Glomeruli were unremarkable (Masson trichrome staining; original magnification, × 200). (**b**) Segmental necrotizing granulomatous arteritis (H&E staining; original magnification, × 400)

A percutaneous kidney biopsy was performed and revealed on light microscopy interlobular and juxtaglomerular noncaseating granulomatous arteritis (Fig. 2), accompanied by severe interstitial inflammation. The glomeruli appeared unremarkable. Immunohistochemistry for IgG, IgA, IgM, C3, C4, kappa and lambda chains was negative. Stainings for fungi and acid-fast bacilli were also negative. Acute segmental and focal granulomatous arteritis induced by ipilimumab was diagnosed.

Antinuclear antibodies and anti-neutrophil cytoplasmic antibodies (ANCA) were negative (anti-MPO <  3.2 and anti-PR3 <  2.3). Angiotensin convertase enzyme (45 UI/L), calcemia (2.24 mmol/L) and chest CT-scan were normal. These negative exams and the absence of cutaneous or peripheral lymph node involvement allowed us to exclude sarcoidosis.

Ipilimumab was immediately discontinued, and the patient was treated with oral prednisone at a dose of 1 mg/kg/day for one month, followed by gradual discontinuation over 4 weeks. Serum creatinine rapidly improved to 2.8 mg/dl two weeks after steroid introduction. One month after steroid discontinuation, serum creatinine remained stable (2.8 mg/dl - Fig. 1). Unfortunately, the patient died a few months later from progression of his melanoma.

## Discussion and conclusions

We report here the first case of kidney vasculitis induced by ICPIs. According to the Chapel Hill International Consensus Conference (CHCC) nomenclature, vasculitides are classified based on vessels size into large (aorta and its main branches), medium (coronary, mesenteric, intra-renal), and small (arterioles and capillaries) vessel disease [6]. Renal small-vessel vasculitides, with or without granuloma, have been described in Giant Cell Arteritis (GCA), polyarteritis nodosa [7], sarcoidosis [8] or ANCA-associated vasculitis. Renal acute necrotizing arteritis with granulomatous perivascular infiltrate has also been reported in a patient with metastatic melanoma treated with encorafenib (BRAF inhibitor) and binimetinib (MEK inhibitor) [9].

ICPIs-related vasculitis, predominantly large-vessel vasculitis and vasculitis of the nervous system, have already been described. Daxini et al. reported 20 cases of vasculitis associated with ICPIs, involving small (digital or retinal vasculitis, granulomatosis with polyangiitis), medium (nervous system) or large vessels (giant cell arteritis), but no case of renal granulomatous arteritis [10]. Goldstein et al. reported two cases of polymyalgia rheumatica and giant cell arteritis (PMR/GCA) following a treatment by ipilimumab [11]. These cases suggest that lymphocyte T costimulation blockers, such as abatacept, may have beneficial therapeutic implications in the management of PMR/GCA.

Cortazar et al. reported kidney pathology in 13 patients with ICPIs-induced acute kidney injury (AKI). Kidney biopsies revealed acute interstitial nephritis (AIN) in 12 patients, with granulomatous features in 3 patients, whereas the last patient presented acute thrombotic microangiopathy (TMA) without AIN [3]. Many cases of AIN have been reported [12, 13], while immune-related glomerulonephritis [4] or nephrotic syndrome [5] have occasionally been described. To our knowledge, we report here the first case of renal granulomatous arteritis induced by ipilimumab.

PD-1 and CTLA-4 are known as immune checkpoints. They prevent activation of T-lymphocyte by binding their respective receptors on T-cells. Cancer cells overexpress PD-1 and/or CTLA-4 proteins to down-regulate T-cells and allow tumor growth and metastasis. Checkpoint inhibitors are monoclonal antibodies (anti-PD-1 and anti-CTLA-4) that are used as an immunotherapy to destroy cancer cells by allowing T-cells to proliferate within the tumor microenvironment. Two mechanisms are proposed to explain the pathophysiology of AIN in patients exposed to ICPIs. First, ICPIs may disrupt tolerance to exogenous drug antigens, allowing reactivation of memory T-cells previously primed by exogenous drug exposure. Second, as the CTLA-4 and PD-1 pathways are normally employed to limit unwanted autoimmunity, interference with CTLA-4 and PD-1 pathways may disrupt peripheral tolerance of self-antigens by blunting activation of self-reactive T-cells. These two pathways may lead to migration of effector T-cells into the kidney and development of acute tubulointerstitial nephritis [14]. Our patient did not take any other drug. So, the second pathway can presumably be involved in vascular T-cells influx leading to development of granulomas in our patient.

Interestingly, single nuclear polymorphisms of *CTLA4* have been reported to be associated with ANCA-positive small vessels vasculitis (AAV) [15]. These genetic polymorphisms may lead to hyperreactivity of T cells and thus contribute to the pathogenesis of AAV [16]. Even though ANCA were negative, we may suppose that pathophysiology of renal granulomatous arteritis in our patient was directly linked to CTLA-4 blocking.

Management of ICPIs-induced AKI is still challenging. The efficacy of glucocorticoids in the treatment of AIN remains controversial in the absence of randomized trials. However, based on case reports and case series, Izzedine et al. suggest that management of immune-related renal toxicities should consist in prompt treatment discontinuation and steroid therapy [2]. In their study, Cortazar et al. reported 2 total remissions (serum creatinine < 0.35 mg/dl above baseline value) and 7 partial remissions (serum creatinine > 0.35 mg/dl above baseline value) among 10 patients receiving a steroid treatment [3]. In our patient, ipilimumab discontinuation and steroid therapy led to partial remission, with nevertheless tumor progression leading to death in the absence of effective substitution treatment for advanced melanoma. This highlights another important consideration, which is the possibility to continue or reintroduce ICPI therapy in patients with kidney injury. In their series, Cortazar et al. reported one patient with relatively stable renal function despite continuation of ipilimumab without steroid therapy [3]. Izzedine et al. suggest the possibility to continue ICPI in case of a grade 1 renal event and to postpone the treatment until plasma creatinine decreases to at least grade 1, in case of grade 2–3 renal event [2].

Finally, whether patients with IRAEs have a better anti-tumor response to ICPI therapy remains unknown. A recent study suggests that IRAEs may be associated with improved progression-free survival (PFS) in patients receiving anti-PD-1/PD-L1 therapy. Furthermore, in this study, the use of systemic corticosteroids does not appear to alter PFS [17].

In conclusion, we report here the first case of ipilimumab-induced renal granulomatous arteritis, a new entity of ICPIs’ immune related adverse events. This case highlights the importance of rapid kidney biopsy to elucidate the cause of kidney injury related to ICPIs and promptly start steroid therapy.

## Data Availability

Further clinical data and images to support this case are available from the corresponding author upon reasonable request.

## Abbreviations

AAV: ANCA-positive small vessels vasculitis
AIN: Acute Interstitial Nephritis;
AKI: Acute Kidney Injury
ANCA: Anti-Neutrophil Cytoplasmic Antibodies
CTLA-4: Cytotoxic T-Lymphocyte Antigen-4
GCA: Giant Cell Arteritis
ICPIs: Immune Checkpoint Inhibitors
IRAE: Immune-Related Adverse Events
PD1: Programmed cell Death protein-1
PDL-1: Programmed cell Death protein-Ligand 1
PMR/GCA: Polymyalgia Rheumatica and Giant Cell Arteritis
TMA: Thrombotic Microangiopathy
